# An In Vitro Evaluation Study of the Geometric Changes of Root Canal Preparation and the Quality of Endodontic Treatment

**DOI:** 10.1155/2020/8883704

**Published:** 2020-08-12

**Authors:** Svetlana Razumova, Anzhela Brago, Ammar Howijieh, Haydar Barakat, Ashot Manvelyan, Yuliya Kozlova

**Affiliations:** Department of Propaedeutic of Dental Diseases, Medical Institute, Peoples' Friendship University of Russia (RUDN University), Moscow 117198, Russia

## Abstract

**Introduction:**

The geometry of root canals differs in different parts, especially in the apical region, and it is affected by different preparation techniques. The aim of this study was to evaluate the geometric changes of root canal preparation by general dentists regardless of the endodontic instrumentation systems and to study the quality of endodontic treatment by evaluating the untouched areas after mechanical preparation and the smear layer removal.

**Materials and Methods:**

100 extracted maxillary canines were collected for the in vitro study from 10 dentists, and the dentists were asked to treat the teeth endodontically. The teeth then were separated and examined under an optical microscope to evaluate the root canal final diameter and the untouched areas. Then, the teeth were examined under a scanned electronic microscope to evaluate the smear layer in coronal, middle, and third parts of the canal. Statistical significance was set as *P* < 0.05.

**Results:**

The mean diameter of the root canal after instrumentation in the coronal and middle thirds was 2.50 ± 1.12 and 1.75 ± 1.24 mm, respectively, and the untouched area percentage observed in the apical thirds was 71%. For smear layer removal, it was better in the coronal and middle thirds than in the apical (*P* < 0.05).

**Conclusion:**

The changes in the diameter of the root canal, the percentage of untouched areas after mechanical preparation, and the percentage of smear layer were observed in a higher percent in the apical third than in the coronal and middle thirds, and this raises the question of changing the technique of processing the root canal, especially in the apical third.

## 1. Introduction

Successful endodontic treatment is based on two basic principles: adequate treatment of the root canal with adequate diagnosis (vital and nonvital pulp) and high-quality mechanical preparation of the root canal with maintaining a minimal apical diameter. The geometry of root canals differs in different parts, especially in the apical region, and it is affected by different preparation techniques [1].

The purpose of mechanical root canal preparation is to make the shipping convenient for irrigation and obturation. The use of rotary instruments in most cases gives the canal a round shape in the coronal and middle thirds, where the taper of the instruments is high. The mechanical treatment removes the dentine from inside the root canal, which changes the geometric dimension of the root canal system [2, 3]. These geometric changes were observed to be higher in the coronal and middle regions of the root canal than in the apical one, which could be related to the greater taper of the instruments [4–6]. From a clinical overview, this increase in the canal diameter in the coronal and middle thirds could improve the reach of irrigation solutions to the apical third which can improve the removal of infected dentine and the smear layer due to the reason that the apical third in some cases may not be prepared by mechanical instruments as effectively as the other parts [5, 7].

An important aspect in endodontic treatment is the untouched walls of the root canal after mechanical preparation, which was observed in a range of 2.6% up to 80%, and this demonstrates that no system or technique could touch all the walls of the root canals [8–11].

Another important aspect is the final diameter of the root canal, and several studies have shown that the final diameter of the tools used for mechanical preparation varied from a diameter of 25 to 40 mm, with a diameter of 30 being the most common final diameter [12].

The third important aspect which decreases the rates of successful endodontic treatment is the smear layer which forms during mechanical instrumentation of the root canal and covers the canal walls. This smear layer contains microorganisms, necrotic tissues, and dentinal remnant and constitutes an obstacle to the delivery of irrigants and chemical agents to the root canal system and creates a barrier between the root canal surfaces and filling materials. Removal of the smear layer and debris provides better sealing of filling materials to root canal surfaces [13, 14].

The aim of this study was to evaluate the geometric changes of root canal preparation by general dentists regardless of the endodontic instrumentation systems and to study the quality of root canal instrumentation by evaluating the untouched areas after mechanical preparation and the smear layer removal.

## 2. Materials and Methods

This in vitro study was conducted in two parts. The study was conducted in accordance with the Helsinki Declaration of 1975, as revised in 2000, and was approved by the Ethical Committee of the Peoples' Friendship University of Russia (Protocol 6 at 21.02.2019).

### 2.1. First Part: Root Canal Preparation

One hundred maxillary canines, extracted for periodontal diseases, were collected from 10 dentists (specialty: dentistry general practice) in Moscow, by 10 teeth from each dentist.

The disadvantage of the proposed method was the inability to measure the diameter of the channel prior to preparation along the entire length. Indirect measurements were carried out visually at the apical foramen when passing the initial file through the coronal third. All teeth had an initial file greater than 15 but less than 30 with a mean diameter of 0.30 ± 0.05 mm.

Dentists were asked to provide instrumentation and irrigation of the root canals according to the protocol they used in their clinic. The most common protocol was using manual instruments (K-file, H-file) (Mani®, INC, Japan) to master file 35, followed with rotary instruments using most common systems: profile (Dentsply® Sirona, USA), MTWo (VDW®, Germany), and Protaрer (Dentsply® Sirona, USA). The size of the last file was 06–30 for Profile, 25/.06 for MTWo, and F2 for Protaper.

Dentists were asked to use the irrigation protocol as follows: sodium hypochloride nitrite 3% (Omega dent, Russia) with ultrasound activation after each file (Woodpecker, China) with endodontic tips E1, followed with EDTA 17% (META® Biomed, Korea), and finally irrigation of the canal with water.

After the treatment, teeth were placed in the saline and moved to the technical laboratory for microscope scanning. The teeth were sagittally separated and examined using an optical microscope (Nikon SMZ-25, Japan) to determine the root canal diameter in coronal, middle, and apical thirds and to determine the untouched areas. The diameter of the root canal was measured and recorded in microns. The untouched area was recorded in coronal, middle, and apical thirds of the root canal.

### 2.2. Second Part of In Vitro Study: Smear Layer Removal

The teeth were examined under a scanned electronic microscope (Vega 3 SB, Tescan Orsay Holding, Kohoutovice, Czech Republic). Samples were dried and coated with platinum using a sputter coater (AJA ORION 8, AJA International INC, California, USA), and the samples were scanned by SEM at a magnification of 10,000x, 25,000x, and 100,000x for each (coronal, middle, and apical) third of the root canal. The evaluation of SEM micrographs was carried out by two examiners in a single-blind assessment. The evaluation of smear layer removal was recorded according to Hülsmann et al. [15] as the following scores:

- Score 1: absence of the smear layer and open dentinal tubules
- Score 2: a small amount of the smear layer covering the root canal and many dentinal tubules are open
- Score 3: a smeared layer covering the walls of the root canal; some dentinal tubules are opened
- Score 4: the surface of the root canal is completely covered with a smear layer; the dentinal tubules are not opened
- Score 5: a heavy smeared layer and debris cover the surface of the root canal

### 2.3. Statistical Analysis

IBM SPSS Statistics v 22.0 licensed package (IBM, Chicago, IL, USA) was used for statistical processing of the study data. The method of descriptive statistics was used for statistical processing of the received data. The comparison of smear layer removal between different thirds of the root canal was tested using Kruskal–Wallis *H* and Mann–Whitney *U*. Statistical significance was set as *P* < 0.05.

## 3. Results

### 3.1. The Root Canal Diameter after Endodontic Treatment, Untouched Areas, and Smear Layer Removal

**Figure 1:**
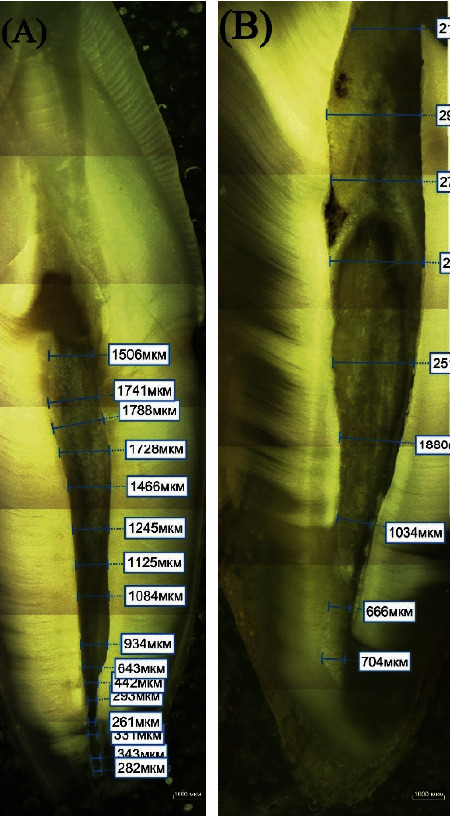
Measuring the diameter of root canals using an optical microscope, showing the differences in the root canal diameter in coronal, middle, and apical thirds.

When analyzing the root canal diameter after preparation with various rotary instruments, it was established that all dentists treated the coronal part of the root canal from 1400 mcm to 3500 mcm and in the middle third, 1200–2000 mcm, and in the apical third, 212–650 mcm (Table 1) (Figure 1).

The apical third of the canal in 70% of the cases was not sufficiently processed.

**Figure 2:**
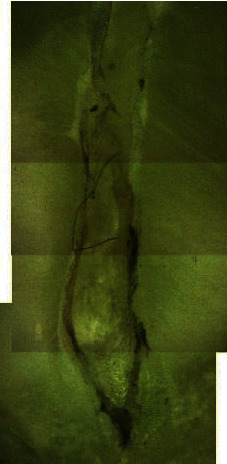
Untouched areas in the apical third of the root canal.

In the majority of the cases, the untouched areas were detected in the apical parts of the root canals (71% of the samples). In the apical third at a level of 3–5 mm from the apex, untreated canal walls were revealed, and the shape did not match the round shape of the instrument (Table 1) (Figure 2).

**Figure 3:**
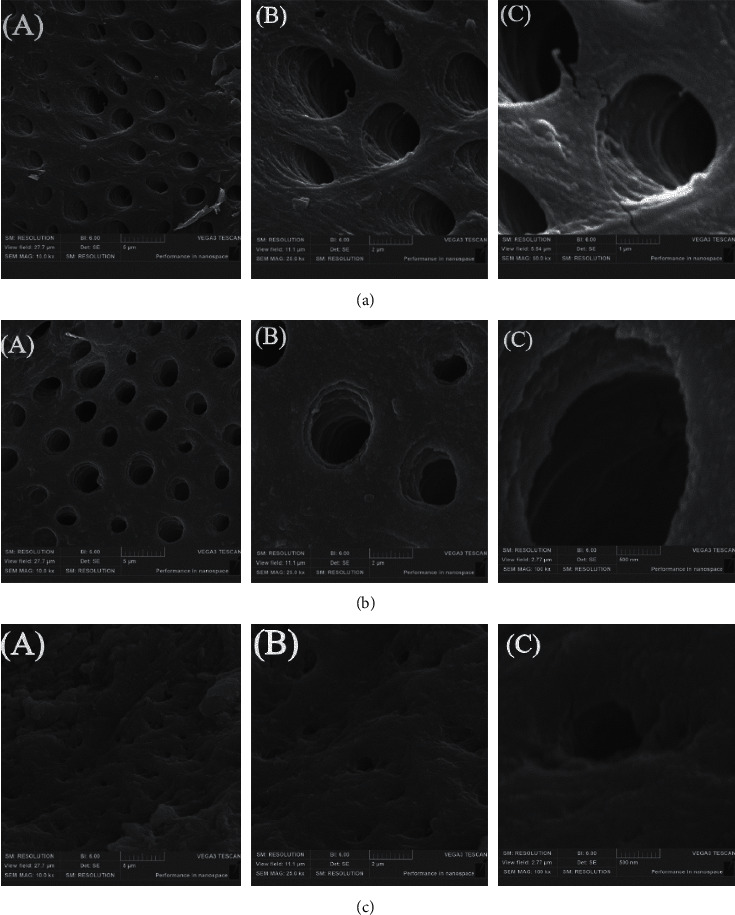
SEM of smear layer removal in (a) coronal, (b) middle, and (c) apical thirds of the root canal. ((A) SEM (×10,000), (B) SEM (×25,000), and (C) SEM (×100,000)).

Regarding the smear layer removal, the specimens showed a higher removal of the smear layer in coronal and middle thirds than in the apical thirds (*P* < 0.05) (Figure 3). The results showed score 1 in 90% and 81% in the coronal and middle thirds, respectively (Table 2).

## 4. Discussion

Endodontic treatment is one of the most conservative treatments in dentistry, and to make it a successful one, many factors should be taken into consideration. Among these factors, a significant knowledge of the root canal system (anatomy, morphology, and shape) and a good mechanical preparation with drug treatment of the root canal decrease the percent of the untouched areas. In this study, we evaluated the preparation of the root canal from different aspects: firstly, we evaluated the final diameter of the root canal after preparation in three thirds by general dentists regardless of the endodontic instrumentation systems and the percent of unprepared areas; secondly, we evaluated the smear layer removal.

In this study, the diameter of the root canal was measured after mechanical and drug treatment using an optical microscope. In the coronal third, the diameter after treatment was 1.4–3.5 mm, and in the middle third of the canal, 1.2–2 mm. In these areas, there was a change in the shape of the root canal compared to the original, especially in the coronal part. The apical orifice in each case was prepared on the apical master file No. 35. When analyzing the diameter of the tooth in the apical third of the canal, zones of untouched root canal walls were observed. When measuring the diameters of the apical part of the canal, variable measurement data were established, when the diameter of the apex changes from smaller to larger. So, in the apical part, a diameter of 585 microns was recorded when preparing this part with an instrument of 350 micron diameter. Another aspect is that, in the apical third, a diameter of 683 microns was recorded, which was not prepared by either manual or rotary tools, and this could be related to the anatomical shape of the root canal. In our opinion, the question how the apical third of the canal is prepared and what shape is given to the canal in the apical third remains completely unstudied. The primary shape of the root canal in the apical third can be different: round, oval, and ribbon (with or without isthmus). Tactile sensations of preparation in the apical third are possible only when using a manual tool. But, the transition to rotary tools with the minimum file sizes (15 and 20) does not provide tactile feedback when preparing the apical third. Regarding the final root canal diameter, a study has recorded the final diameter of mesial canals of mandibular molars after preparation at 1 mm from the apex varied between 0.28 and 0.40 mm, and these results coincide with our results [16].

Regarding the untouched areas, they were observed in the apical third even though the canal was processed by irrigation of sodium hypochlorite 3% with the activation of ultrasound. Studies have demonstrated that untouched walls exist in the first place in areas with anatomical complex structures such as the isthmus, grooves, and flattened root canals. This structure can serve as a potential risk for infection due to the existing microorganisms and infectious dentine [17–19]. Studies have demonstrated that untouched areas can be reduced by increasing the apical preparation size and that also could reduce the intracanal bacteria [20–22].

Changes in the root canal geometry could be due to the changes in the morphology of the teeth, the used tools, or the evaluation methodology used. According to different studies, the percentage of untouched walls ranged from 8.17% to 58.8% [12, 23] for the entire canal in groups of teeth with flattened shape canals and from 3.13% to 51.03% in the apical area [24, 25]. The presence of untouched walls might be explained by the variability of the design of tools such as taper, diameter, and cross section [26, 27].

In 2009, Paqué et al. conducted a research to study the effectiveness of root canal treatment on the geometry of the apical part using micro-CT. The study was performed on extracted maxillary molars using different systems: FlexMaster, GT Rotary, LightSpeed, ProFile, ProTaper instruments, or nickel-titanium K-files for manual processing. According to the results of the study, there were no differences in the volume of the apical part of the canal before treatment in the experimental groups. The area of untreated canals varied from 4% to 100% and was generally larger in the mesiobuccal and palatal canals than in the mesiodistal canals [1]. A literature review has confirmed that the design of the endodontic instruments and the anatomical structure of the root canal system are the major factors that affect the biomechanical preparation of root canals [28].

It is agreeable that the design of the endodontic instrument affects the root canal preparation and removes a higher percent of the untouched areas; we did not assess the impact of this design on the detection of the untouched areas because recently so many instrument designs were detected, and some of them were designed according to the root canal shape like the XP-endoshaper; the purpose of our study was to determine the quality of root canal instrumentation side by side with the irrigation protocol, and we found that, in some cases, neither the instrument design nor the root canal irrigation can reach every areas, which should promote the manufactures to rethink of modifying the instrument design taking into consideration the anatomy and morphology of the root canal system.

For smear layer removal, using EDTA 17% together with NaOCl 3% in cleaning root canals was effective in coronal and middle thirds for the root canal, and these results agreed with the Spangberg study, which showed that EDTA removes the smear layer effectively from the canal surface [29]. SEM scans in the apical region of the root canal showed a thicker smear layer at different magnifications (Figure 3). Many studies suggest using physical methods such as laser or ultrasound to activate the irrigant inside the root canal and that will greatly remove the smear layer [30, 31].

## 5. Conclusion

Within the limits of this study, existing protocols and methods for root canal treatment do not provide adequate qualitative preparation, especially for the apical third of the root canal. The irrigation protocol does not allow for the qualitative removal of the smear layer in the apical third.

The obtained data raise the question of changing the technique of processing the root canal, especially in the apical third (AMF size and checking the apical preparation by manual techniques using peripheral filing techniques), or changing the irrigation protocol, such as using different protocols for irrigation or combination between irrigants with or without physical activation to achieve a clean environment for obturation.

## Figures and Tables

**Table 1:** The diameter of the root canal and the percentage of the untouched area in coronal, middle, and apical thirds.

Parameter	Coronal third	Middle third	Apical third
Diameter of the root canal (mm)	2.50 ± 1.12	1.75 ± 1.24	0.38 ± 0.08
The percentage of the untouched area	0	0	71

**Table 2:** Scores of smear layer removal in coronal, middle, and apical thirds of the root canal.

Scores	Coronal third	Middle third	Apical third^*∗*^
1	90%	81%	0
2	10%	14%	0
3	0	5%	78%
4	0	0	22%
5	0	0	0

^*∗*^ *p* < 0.05—the level of significance.

## Data Availability

The data used to support this study are available upon request.
